# ATRON: Autonomous trash retrieval for oceanic neatness

**DOI:** 10.3389/frobt.2025.1718177

**Published:** 2026-01-22

**Authors:** John Abanes, Hyunjin Jang, Behruz Erkinov, Jana Awadalla, Anthony Tzes

**Affiliations:** 1 Electrical and Computer Engineering, NYU Tandon School of Engineering, New York, NY, United States; 2 Mechanical Engineering, Virginia Tech, Blacksburg, VA, United States; 3 Electrical Engineering, New York University Abu Dhabi, Abu Dhabi, United Arab Emirates; 4 Mechanical Engineering, Egyptian Refining Company, Cairo, Egypt; 5 Center of Artificial Intelligence and Robotics (CAIR), New York University Abu Dhabi, Abu Dhabi, United Arab Emirates

**Keywords:** collision avoidance, path planning, uncrewed marine vessel, YOLO object detection, orienteering problem

## Abstract

The subject of this article is the development of an unmanned surface vehicle (USV) for the removal of floating debris. A twin-hulled boat with four thrusters placed at the corners of the vessel is used for this purpose. The trash is collected in a storage space through a timing belt driven by an electric motor. The debris is accumulated in a funnel positioned at the front of the boat and subsequently raised through this belt into the garbage bin. The boat is equipped with a spherical camera, a long-range 2D LiDAR, and an inertial measurement unit (IMU) for simultaneous localization and mapping (SLAM). The floating debris is identified from rectified camera frames using YOLO, while the LiDAR and IMU concurrently provide the USV’s odometry. Visual methods are utilized to determine the location of debris and obstacles in the 3D environment. The optimal order in which the debris is collected is determined by solving the orienteering problem, and the planar convex hull of the boat is combined with map and obstacle data via the Open Motion Planning Library (OMPL) to perform path planning. Pure pursuit is used to generate the trajectory from the obtained path. Limits on the linear and angular velocities are experimentally estimated, and a PID controller is tuned to improve path following. The USV is evaluated in an indoor swimming pool containing static obstacles and floating debris.

## 1 Introduction

There are an estimated 269,000 tons of plastic on the water surface (Prakash and Zielinski, 2025). Projections indicate that the global plastic waste production will surpass one billion metric tons (Subhashini et al., 2024). These vast amounts of primarily plastic debris persist in the environment and require hundreds of years for decomposition.

During decomposition, microplastics contain hazardous chemicals and cause lasting damage (Chrissley et al., 2017). Toxins originating from plastic elements disrupt ecosystems and pose threats to human health, including cancers, birth defects, and immune system disorders (Akib et al., 2019).

Traditional labor-intensive methods for waste collection are insufficient due to the scale and dispersed nature of the problem in remote or hazardous locations (Flores et al., 2021), and stationary solutions are hindered by environmental changes (Subhashini et al., 2024). Recently, the focus has shifted from manual cleanup (Chandra et al., 2021) to robotic systems that address ocean pollution (Akib et al., 2019).

Unmanned surface vehicles (USVs) are at the forefront of these efforts (Bae and Hong, 2023; Fulton et al., 2019; Turesinin et al., 2020; Costanzi et al., 2020; Shivaanivarsha et al., 2024; Lazzerini et al., 2024; Suryawanshi et al., 2024); they are designed for collecting floating debris from rivers, ponds, and oceans. Commercial solutions such as WasteShark, Clearbot, and MANTA are used for trash collection.

The development of these robots is underpinned by technologies such as a) edge computing (Carcamo et al., 2024; Chandra et al., 2021; Salcedo et al., 2024), b) computer vision and AI for object classification (Li et al., 2025), c) environmental awareness (Li et al., 2024; Wang et al., 2019), d) intelligent navigation and control (Gunawan et al., 2016; Li et al., 2025; Wang et al., 2019), and e) effective debris collection (Li et al., 2024; Subhashini et al., 2024; Akib et al., 2019).

Marine cleaning robots are often limited by their small-scale and restricted operational capacities (Chandra et al., 2021; Flores et al., 2021). The dynamic nature of aquatic environments poses a complex challenge for effective and adaptable path planning, and operation in remote or GPS-denied areas is particularly difficult (Wang et al., 2019).

Several challenges remain in developing autonomous debris collection systems, including lighting and weather conditions, efficient path planning, robust navigation, and mechanical designs for handling diverse floating waste.

**FIGURE 1:**
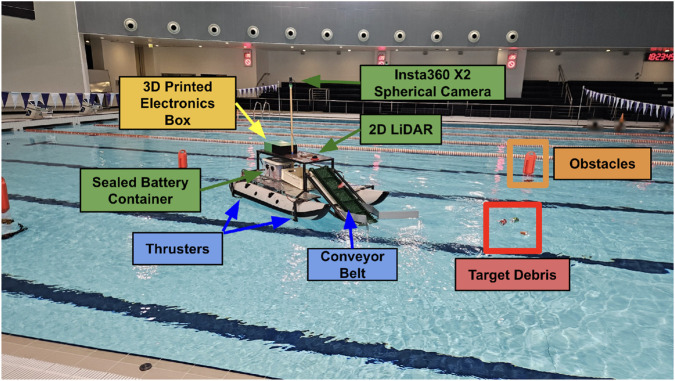
ATRON: an autonomous USV capable of extracting debris from water surfaces.

This article presents Autonomous Trash Retrieval for Oceanic Neatness (ATRON), addressing these challenges through an integrated approach that combines mechanical design, perception, planning, and control, as shown in Figure 1. The designed USV is similar to that in Ahn et al. (2022) and utilizes a robust twin-hulled catamaran design that enables heavy-duty debris collection operations. There are some fundamental changes, including the use of SLAM and various sensors (spherical camera, LiDAR) for USV localization and algorithms related to path planning relying on the orienteering problem and the rapidly exploring random tree (RRT) algorithm.

The contributions in this article include the following:

- The development of a twin-hulled, ROS-based USV, ATRON, for collecting up to 1 ${\text{m}}^{3}$ of floating debris. Four independent thrusters placed at the edges of the vessel provide a linear velocity of up to 1.47 m/s and an angular velocity of up to 0.3 rad/s.
- The development of a visual system for debris and obstacle detection using a spherical camera; YOLOv11 is used for debris classification (Ali and Zhang, 2024).
- The utilization of the orienteering problem for task planning and RRT for obstacle avoidance.
- Simulation and experimental studies conducted in an indoor pool for debris collection and obstacle avoidance.
- The development of a GPU-based physics simulator (Isaac Sim) for the evaluation of several classification algorithms under various sea states and lighting conditions.

## 2 ATRON design (materials and equipment)

The ATRON is a twin-hulled catamaran measuring (2.7×1.5×1.65) m (above the waterline), with a dry weight of 120 kg and a 0.15 m draught depth; and it is stable up to sea-state 2. Two parallel pontoons constitute its framework and are connected via an aluminum ( 1.75×1.2 m) platform, while a secondary elevated ( 1.35×0.6 m) platform is used for its electronics, including the 360 ° camera mounted on top of a 1.2-m pole. The conveyor belt used for trash removal, isolation absorbers, and the four underwater thrusters supplement the structure.

**FIGURE 2:**
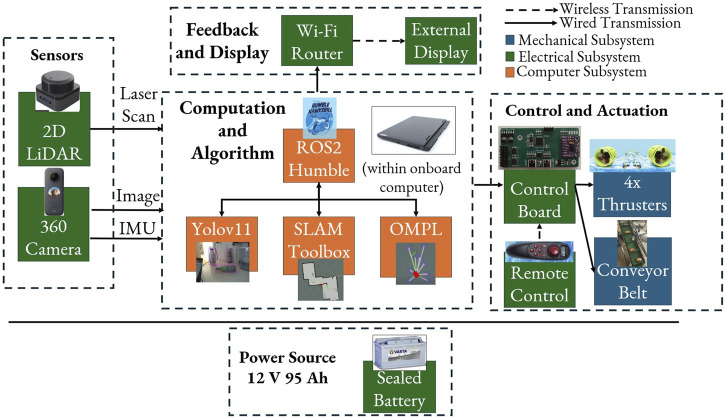
ATRON structural block diagram.

Figure 2 illustrates the comprehensive system architecture integrating the mechanical, electrical, and computational subsystems. The USV operates on a hierarchical control structure built on ROS 2 Humble (Abaza, 2025), enabling modular communication between perception, planning, and actuation components.

The Insta360 × 2 spherical camera provides 360° visual coverage for debris detection, while the Slamtec S2P 2D LiDAR is used for SLAM and obstacle avoidance, and the BNO055 9-DOF IMU is used for attitude estimation and sensor fusion. YOLOv11 (Ali and Zhang, 2024) is used for real-time debris and obstacle detection from the spherical camera feed, within the SLAM Toolbox (Macenski and Jambrecic, 2021) for GPS-denied localization using LiDAR and inertial measurement unit (IMU) fusion, while components of the OMPL (Şucan et al., 2012) are used for path planning. The system can be connected through a Wi-Fi/cellular router.

The ATRON utilizes a differential thrust propulsion system with four brushless thrusters positioned at the vessel’s corners on extended aluminum outriggers. Each thruster delivers 1.2 kgf at 12 V, controlled by bidirectional ESCs capable of 50 Hz PWM modulation for velocity control. A custom controller based on the STM32F103C8T6 microcontroller serves as the interface between high-level ROS 2 commands and low-level actuator control. It provides four PWM ESC-based output channels, PWM control for the conveyor motor via a solid-state relay, and ${\text{I}}^{2}$ C communication with the IMU. The controller communicates with the onboard computer via a USB-to-UART bridge, exchanging JSON-formatted commands and sensor data at 50 Hz for real-time control.

The debris collection system is a 300-mm-wide reinforced rubber timing belt with molded cleats spaced at 50 mm intervals. The belt spans a 600 mm incline from the waterline to the collection container, driven by a 500 W brushed DC motor with integrated 10:1 reduction gearing. Drainage holes (10 mm diameter) are distributed across the belt surface at 30 mm intervals to prevent water accumulation that could reduce lifting capacity or destabilize the collected items. The ATRON has a V-shaped extended metal funnel with a 120 ° opening angle to guide debris toward the collection zone.

**FIGURE 3:**
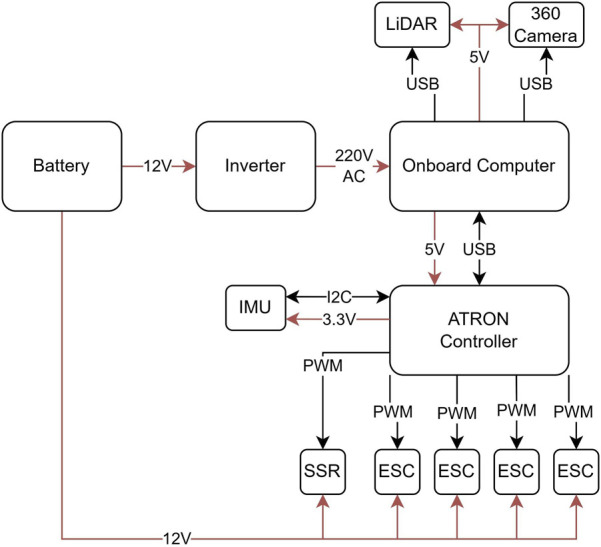
ATRON electrical systems.

All systems operate from a 12 V, 95 Ah sealed waterproof environment lead-acid battery providing 5.84 operating hours using a DC-to-AC inverter unit, while the USB computer-port can provide ample power for the LiDAR, the 360° camera, and the ATRON controller, which uses an onboard voltage regulator powering the IMU-sensor and any other circuitry. The thrusters are powered from bidirectional ESCs, while a solid-state relay is used in the conveyor mechanism, as shown in Figure 3.

The ATRON microcontroller relies on the STMicroelectronics STM32F103C8T6, which provides six PWM output channels using dedicated hardware timers. All PWM signals are level-shifted to 5 V using a TXB0104PWR bidirectional voltage-level translator to ensure compatibility between the MCU and the external devices. The high-speed I^2^C bus is interfaced to a BNO055 IMU. This microcontroller polls the IMU and provides the sensor data in JSON format.

## 3 ATRON software design (methods)

### 3.1 Image rectification

**FIGURE 4:**
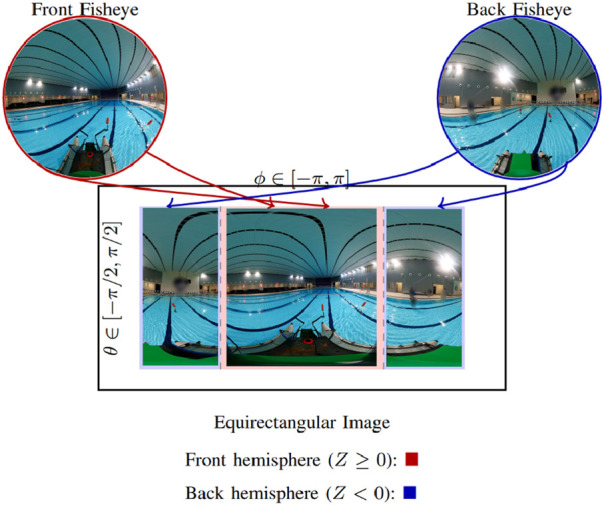
Dual fisheye image to equi-rectangular image.

The 360° spherical camera observes the surrounding space and returns dual fisheye images, as shown in Figure 4. The camera’s extrinsic calibration parameters include a) $c_{x}$ and $c_{y}$ , which correspond to the deviation of each hemispherical image from the optical center; b) crop size; and c) $T={\left[t_{x},t_{y},t_{z}\right]}^{T}$ and $R=R_{z}\left(\psi \right)R_{y}\left(\theta \right)R_{x}\left(\phi \right)$ , determining the relative translation and rotation between the front and back hemispherical images.

After calibration, the equirectangular projection is applied as described by Flores et al. (2024). Each pixel (u,v) in the equirectangular image is first converted to spherical coordinates (ϕ,θ) via Equation 1

$$\phi =\frac{2\pi u}{W}-\pi ,\theta =\frac{\pi v}{H}-\frac{\pi }{2},$$ (1)

where W (H) is the width (height) of the equirectangular image. These are then converted to 3D unit vectors described in Equation 2:

$$p^{⊤}=\left[X,Y,Z\right]=\left[\cos\theta \sin\phi ,\;\sin\theta ,\;\cos\theta \cos\phi \right].$$ (2)

For the front hemisphere points (Z≥0) , the projection is described in Equation 3

$$\begin{array}{ll} & r=\sqrt{X^{2}+Y^{2}},\alpha =\arctan\left(\frac{r}{\left|Z\right|}\right),\\ & \;r_{\text{fisheye}}=\frac{\alpha w}{\pi }, \end{array}$$ (3)

where w is the width of the fisheye image. The pixel coordinates in the front fisheye image are $x_{f}=c_{x}^{f}+\frac{X}{r}\cdot r_{\text{fisheye}},\;y_{f}=c_{y}^{f}+\frac{Y}{r}\cdot r_{\text{fisheye}}.$ A similar approach is used for points in the back hemisphere. The resulting fisheye to equirectangular process is shown in Figure 4.

The equirectangular format enables the extraction of perspective views at arbitrary viewing angles. Given a field of view (FoV), azimuth Θ , and zenith Φ , each pixel (i,j) in the output perspective image is mapped in Equations 4–7:

$$d=\frac{1}{\left\|p\right\|}\left[\begin{array}{c} 1\\ 2\left(i/W_{d}-0.5\right)\tan\left(\text{FoV}/2\right)\\ -2\left(j/H_{d}-0.5\right)\tan(\frac{\text{FoV}H_{d}}{2W_{d}}, \end{array}\right].$$ (4)

$$d^{\prime }=R_{y}\left(-\Phi \right)R_{z}\left(\Theta \right)d={\left[x^{\prime },y^{\prime },z^{\prime }\right]}^{T},$$ (5)

$$u=W\left(\frac{\arctan2\left(y^{\prime },x^{\prime }\right)}{2\pi }+0.5\right),$$ (6)

$$v=H\left(0.5-\frac{\arcsin\left(z^{\prime }\right)}{\pi }\right),$$ (7)

where (u,v) represent the equirectangular coordinates.

**FIGURE 5:**
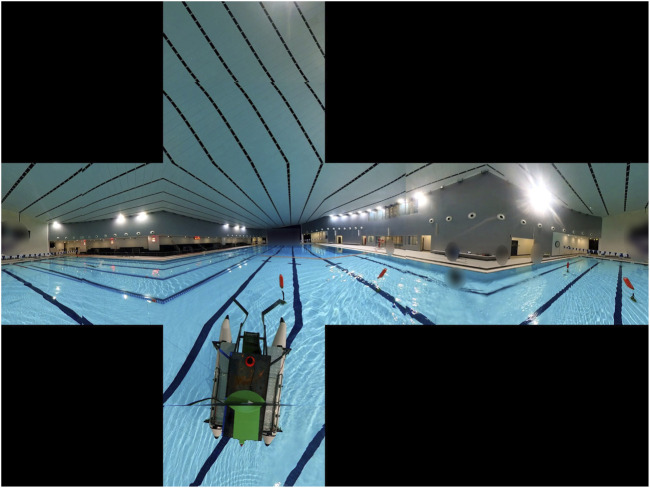
Cubemap representation with FoV 90° .

The camera records (W×H) = (3840×1920) dual fisheye images. The cubemap images in Figure 5 have a FoV of 90° , a width $W_{d}$ of 960, and a height $H_{d}$ of 960 pixels. The generated views of the left, front, up, down, right, and back perspectives, respectively, are shown in Figure 5.

The ATRON utilizes a Slamtec S2P LiDAR (Madhavan and Adharsh, 2019) with a range of 50 m and angular resolution of 0.1125 ° with a 32 KHz sampling rate, along with a BNO055 9-DOF IMU.

### 3.2 2D to 3D object projection

Since the debris floats on the water’s surface, LiDAR cannot be used in wavy sea states, and visual methods are utilized instead, relying on YOLOv11 (Ali and Zhang, 2024). Since soda cans mostly constitute the debris and floatable buoys are the obstacles, the YOLOv11 model was trained on 960×960 images containing objects with these two possible classes.

**FIGURE 6:**
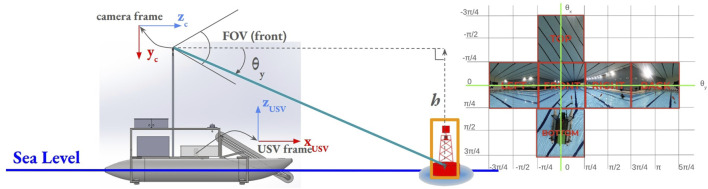
2D image to 3D Cartesian coordinate projection (right). $\theta_{y}$ , the vertical angle between the camera axis and the detected object is calculated using the heading equation for $\theta_{y}$ . $\theta_{x}$ and $\theta_{y}$ are overlaid over the cubemap projection images (right). Notably, calculations are valid only for $\theta_{y}\geq 0,\;h=1.5$ m.

The distance (depth) of the debris or obstacles was estimated, and given that the camera was attached at a given height with respect to the sea level, as shown in Figure 6, a methodology similar to Li et al. (2025) was utilized, assuming that $\theta_{y}$ is positive downward.

The transformation from the 3D-coordinates ${\left[X_{c},Y_{c},Z_{c}\right]}^{⊤}$ in the camera frame to pixel coordinates ${\left[u,v\right]}^{⊤}$ is shown in Equation 8 (Wang et al., 2010)

$$\left[\begin{array}{c} u\\ v\\ 1 \end{array}\right]=\left[\begin{array}{ccc} f_{x} & 0 & c_{x}\\ 0 & f_{y} & c_{y}\\ 0 & 0 & 1 \end{array}\right]\left[\begin{array}{c} X_{c}\\ Y_{c}\\ Z_{c} \end{array}\right],$$ (8)

while the horizontal distance of the object (debris or obstacle) to the camera (Wang et al., 2010) is $Z_{c}=\frac{h}{\tan\left(\theta_{y}\right)}$ .

The heading $\theta_{y}$ (angle between the camera’s principal axis and the ray connecting the camera to the object) is $\theta_{y}=\frac{\text{FoV}}{2}\frac{\left(v-c_{y}\right)}{f_{y}}.$ The yaw angle of the object in the camera’s x-axis, $\theta_{x}$ , is described in Equation 9

$$\theta_{x}=\frac{\text{FoV}}{2}\frac{\left(u-c_{x}\right)}{f_{x}},\text{ while }X_{c}=Z_{c}\tan\left(\theta_{x}\right).$$ (9)

### 3.3 USV-kinodynamics

Assuming minimal roll and pitch of the USV, its state vector x in a 2D environment (Fossen, 2021) is $x={\left[x,y,\theta ,v,\omega \right]}^{⊤}$ , where a) x,y are USV’s position coordinates in the 2D plane, b) θ is the USV’s heading angle, c) v is the linear velocity in the direction of the USV’s heading, and d) ω is the USV’s angular velocity around the vertical axis. The control input vector commands are $u={\left[T,\tau \right]}^{⊤}$ and affect the USV’s speed and turning rate; T corresponds to the thrust generated by the propulsion system, and τ is the USV’s rotational torque that affects its heading.

The USV’s differential kinematics is $\dot{x}=v\cos\theta ,\;\dot{y}=v\sin\theta ,\;\dot{\theta }=\omega ,$ while its simplified hydrodynamics model is expressed in Equation 10

$$\dot{v}=\frac{1}{m}\left(T-D_{v}v\right),\;\dot{\omega }=\frac{1}{I}\left(\tau -D_{\omega }\omega \right),$$ (10)

where m(I) is the USV’s mass (moment of inertia around the Z -axis) and $D_{v}\left(D_{\omega }\right)$ is the linear (angular) drag coefficient.

### 3.4 USV simultaneous localization and mapping

The USV utilizes 2D LiDAR complemented by its IMU to perform SLAM. The orientation data from the IMU are fused to improve the accuracy of the SLAM algorithm using the extended Kalman filter (EKF) (Ribeiro, 2004; Einicke and White, 2002). The EKF recursively estimates the robot state $x_{k}={\left[x,y,\theta \right]}^{T}$ by fusing the planar odometry from scan matching with IMU orientation measurements. The filter’s prediction step is expressed by Equations 11, 12

$${\hat{x}}_{k|k-1}=f\left({\hat{x}}_{k-1|k-1},u_{k}\right),$$ (11)

$$P_{k|k-1}=F_{k}P_{k-1|k-1}F_{k}^{T}+Q_{k},$$ (12)

followed by its update step in Equations 13–15.

$$K_{k}=P_{k|k-1}H_{k}^{T}{\left(H_{k}P_{k|k-1}H_{k}^{T}+R_{k}\right)}^{-1},$$ (13)

$${\hat{x}}_{k|k}={\hat{x}}_{k|k-1}+K_{k}\left(z_{k}-h\left({\hat{x}}_{k|k-1}\right)\right),$$ (14)

$$P_{k|k}=\left(I-K_{k}H_{k}\right)P_{k|k-1},$$ (15)

where $F_{k}$ and $H_{k}$ are the Jacobians of the motion and measurement models, respectively, $Q_{k}$ and $R_{k}$ represent process and measurement noise co-variances, respectively, and $K_{k}$ is the Kalman gain. The covariance of the LiDAR (IMU) odometry is obtained from its specifications.

### 3.5 Orienteering problem for USVs

The order of the collected debris is optimized by treating each piece of debris as a node in a classic orienteering problem (OP). Each debris node is considered to have a score $S_{i}$ , and the edge joining nodes i and j are $E_{ij}=\left\{0,1\right\}$ , where $E_{ij}=1$ signifies that the edge between nodes i and j is chosen as part of the optimized orienteering cycle. The goal is to maximize the ensuing objective function (Gunawan et al., 2016) in Equation 16.

$${\text{argmax}}_{i,j}\overset{N-1}{\underset{i=2}{\sum }}\overset{N}{\underset{j=2}{\sum }}S_{i}E_{ij}.$$ (16)

Let $d\left(E_{ij}\right)$ be the length of the edge $E_{ij}$ ; then, OP satisfies the constraint in Equation 17

$$\overset{N-1}{\underset{i=1}{\sum }}\overset{N}{\underset{j=2}{\sum }}d\left(E_{ij}\right)E_{ij}\leq c,$$ (17)

where c is the maximum length of the orienteering cycle.

**FIGURE 7:**
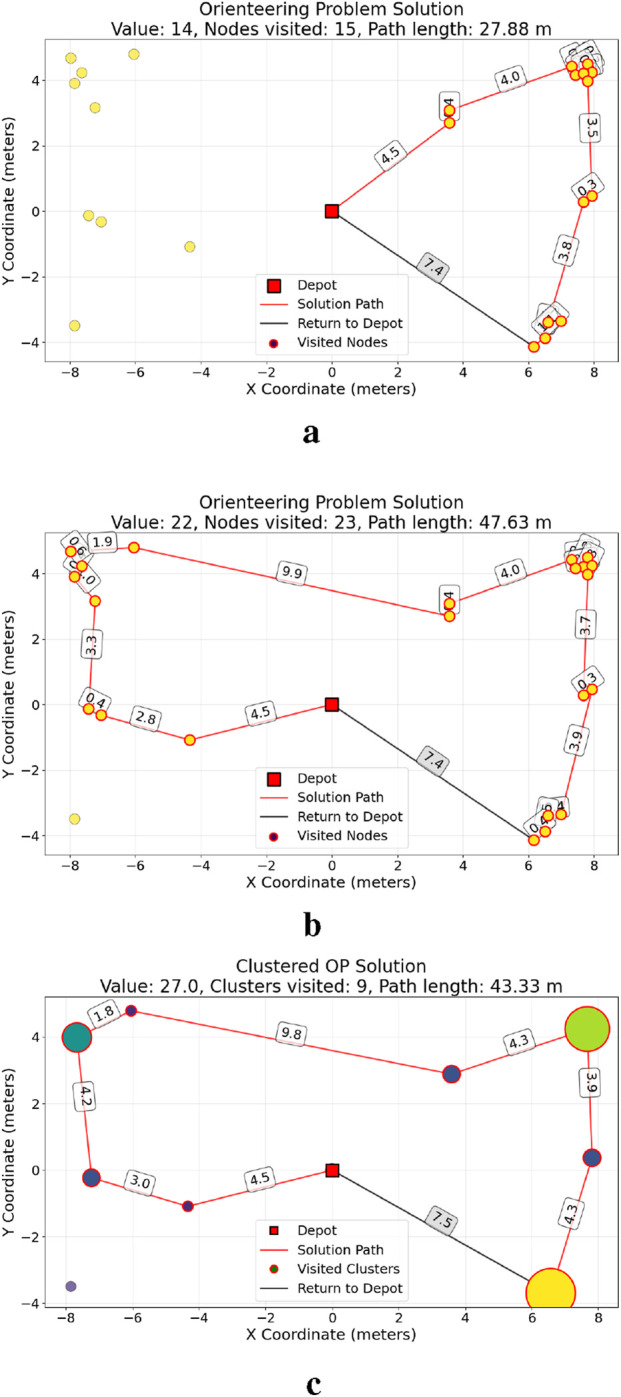
OP solution for various distance constraints. **(a)** OP solution with c = 30 m, **(b)** OP solution with c = 50 m, **(c)** Clustered OP solution via heuristic approach for c = 50 m. **(a, b)** Unclustered output; **(c)** clustered nodes.

The USV implements a heuristic approach (Gunawan et al., 2016) to the OP. Figure 7 indicates that the orienteering path varies depending on the cost c∈{30,50} meters, where the depot node is at (0,0).

The adopted approach assumes static debris, and a static high-level global planner is utilized, followed by a dynamic low-level local planner for small debris using a clustered OP (Angelelli et al., 2014; Elzein and Caro, 2022). A greedy algorithm is introduced that creates clusters with a maximum diameter, which represents regions in which the low-level dynamic follower is implemented.

Given nodes N with positions and the maximum diameter $d_{\max}$ , clusters are produced satisfying their maximum cluster diameter constraint $\text{diameter}\left(C_{j}\right)=\max_{n_{i},n_{k}\in C_{j}}\|p_{i}-p_{k}{\|}_{2}\leq d_{\max},$ where $p_{i}$ is the position of node $n_{i}$ .

A greedy algorithm selects a random node to be a cluster and then inspects each unassigned node n* . If including n* results in a cluster with a smaller diameter than $d_{\max}$ , then it is included in that cluster. The algorithm continues until no further clusters can be created and has a complexity of $O\left(N^{2}\right)$ .

Each cluster’s position is its centroid, and its score equals the number of nodes it contains. The original orienteering solution path is then mapped through these clusters, with consecutive duplicate clusters removed, as shown in Figure 7C. This enables hierarchical planning, in which global routes are determined using clusters, while local control manages individual debris.

This allows the planning algorithm in Section 3.6.1 to be agnostic to small deviations in the position of the debris (because of sea currents).

### 3.6 Path and trajectory planning

#### 3.6.1 Path planning via OMPL

For path planning and obstacle avoidance, the USV relies on the OMPL (Şucan et al., 2012), and the USV’s footprint (L×W) = ( 2.7×1.5 m) is utilized. The USV’s configuration space is determined by extracting the free space obtained from the SLAM algorithm and subtracting the obstacle space. The path of the USV is then constrained by supplying the OMPL’s constrained planner (Kingston et al., 2019). The USV can be thought of as a tank-driven system represented by Wang et al. (2019), where $v_{L}\;\left(v_{R}\right)$ is the velocity created by the left (right) thruster side described by Equation 18.

$$\left[\begin{array}{c} \dot{x}\\ \dot{y}\\ \dot{\theta } \end{array}\right]=\left[\begin{array}{cc} \frac{1}{2}\cos\theta & \frac{1}{2}\cos\theta \\ \frac{1}{2}\sin\theta & \frac{1}{2}\sin\theta \\ -\frac{1}{W} & \frac{1}{W} \end{array}\right]\left[\begin{array}{c} v_{L}\\ v_{R} \end{array}\right].$$ (18)

These constraints are used in OMPL to obtain the path P={p|p=(x,y,θ)} via the RRT connect algorithm (Karaman and Frazzoli, 2011; Kuffner and LaValle, 2000). In environments with sparse obstacles, paths between subsequent nodes are obtained within less than 2 s on average as the USV can, in the vast majority of cases, simply drive around the obstacle.

#### 3.6.2 Path tracking

The velocity of the USV is calculated using a regulated pure pursuit (RPP) controller (Macenski et al., 2023), an improved implementation of the classic pure pursuit (PP) controller, which operates at a constant linear velocity. The classic PP controller follows a look-ahead point specified by the look-ahead radius R . The furthest point on the desired path within this look-ahead radius is considered. The PP algorithm geometrically determines the curvature required to drive the vehicle from its current position to the look-ahead point. The curvature κ is $\kappa =\frac{2\Delta x}{R^{2}}$ , where Δx is the lateral offset of the look-ahead point in the vehicle’s coordinate frame, and R is the look-ahead radius. The vehicle’s coordinate system is placed at the rear differential with the x -axis aligned with the vehicle’s heading. The USV operates with a constant linear velocity v (except for the starting and end points). The required angular velocity is $\omega =\kappa \cdot v=\frac{2\Delta x\cdot v}{R^{2}}.$ The look-ahead distance R acts as a tuning parameter: larger values result in smoother tracking with less oscillation but slower convergence to the path, while smaller values provide a tighter path following at the cost of potential oscillations.

In RPP, the linear velocity is further scaled to improve performance along tight turns. Let v be the desired nominal speed. Let $r_{m}in$ be the minimum radius of curvature that the USV can turn at this given nominal speed. RPP reduces the commanded linear velocity based on a threshold curvature $\kappa_{\text{thresh}}$ . The implementation utilizes a modified regulated pure pursuit (MRPP) controller that has a final threshold $\kappa_{\text{max}}$ , which acts as a safety net to calculate the linear velocity $v_{\kappa }$ in Equation 19.

$$v_{\kappa }=\left\{\begin{array}{cc} v,\; & |\kappa |<\kappa_{\text{thresh}}\\ v\frac{\left|r_{\min}\right|}{\left|\kappa \right|},\; & \kappa_{\text{thresh}}\leq |\kappa |<\kappa_{\text{max}}\\ 0\; & \kappa >\kappa_{\text{max}} \end{array}\right.$$ (19)

**FIGURE 8:**
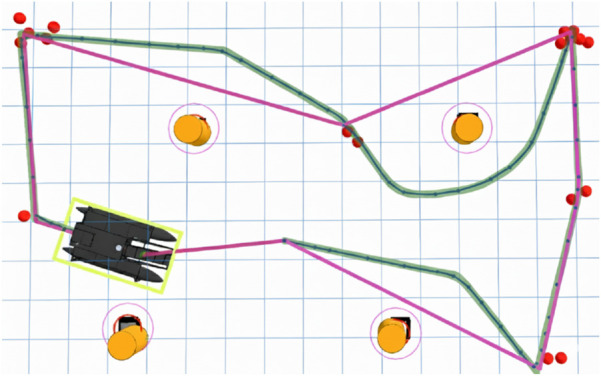
Orienteering (purple), OMPL (green), and actual (blue) path of the USV.

The MRPP was used to follow the path generated by OMPL shown in Figure 8. The USV’s actual path is represented by the blue line using R=0.4 m, $r_{\text{min}}=1.0$ m, and v=1.47 m/s.

#### 3.6.3 PID control and signal mixing

In order to achieve the desired behavior, the input velocity of the USV is sent to a PID controller. PID controllers are used for the linear (angular) USV velocity. Discrete PID-controllers are then utilized with a $T_{s}$ -sampling period.

## 4 Results

### 4.1 Camera calibration

The camera’s extrinsic parameters are estimated by performing live dual-fisheye to equirectangular mapping in order to minimize any stitching artifacts, as shown in Figure 4. The camera uses 1920×1920 pixel-images, resulting in the following intrinsic and extrinsic parameters from Wang et al. (2010), as summarized in Table 1.

**TABLE 1:** Fisheye camera configuration parameters.

Parameter	Value
Crop diameter	1,920 pixels
Output dimensions	3,840×1,920 pixels
Extrinsic translation T	${\left[0.0,0.0,-0.105\right]}^{T}$ m
Extrinsic rotation R	${\left[-0.5{}^{\circ},0.0{}^{\circ},1.1{}^{\circ}\right]}^{T}$
Focal length $f_{x}\;\left(f_{y}\right)$	509.75 (509.75) pixels
Principal point $\left(c_{x},c_{y}\right)$	(960,960) pixels
Distortion coefficients k	[0.146, 0.037, 0.018,−0.010]

Using these parameters allows the formation of equirectangular images in 49 m and the cubemap images in 79 m, resulting in a total per-image processing time of 128 m at 4K resolution when deployed on an Intel i5 processor.

### 4.2 Object detection results

**FIGURE 9:**
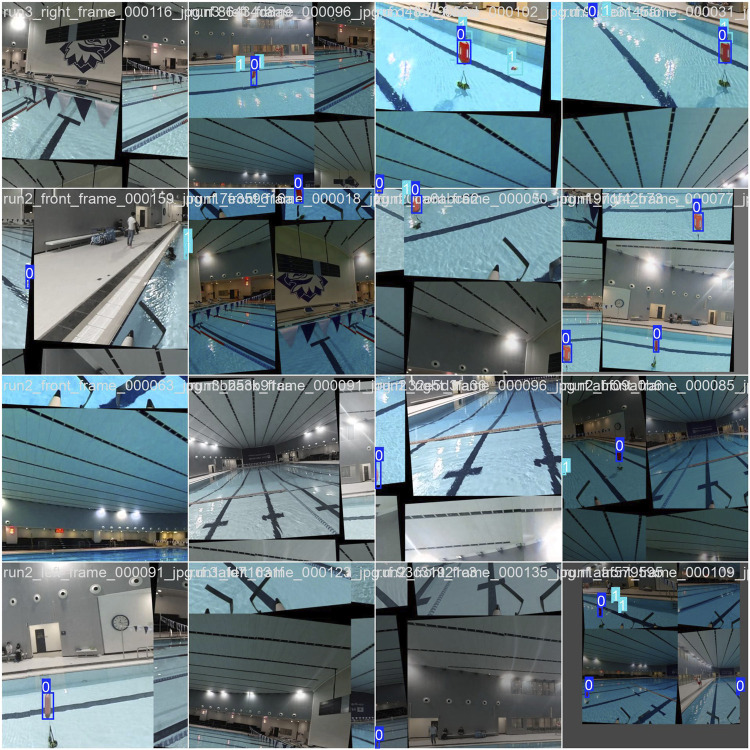
Preprocessed images in YOLO training.

Debris (obstacles) such as cans/plastic bottles (buoys) were collected by recording 3,840×1,920 images from the 360° camera and extracting four 960×960 cubemap images representing the front, left, right, and back image views. A total of 1,416 images were extracted and annotated. Six image-augmentation steps were applied, including a) horizontal flipping, b) ±10% rotation, c) ±25% saturation, d) ±10% exposure, e) random bounding box flipping, and f) mosaic stitching, as shown in Figure 9. These augmentations increased the training data size to 5,192 images. Training–validation–test split was divided in a 70–20–10 ratio.

Varying lighting conditions were used by adjusting the image brightness and saturation. Cubemap face images were also rotated to make the model more adaptable against USV tilt/yaw, and mosaic stitching was implemented. These preprocessing adjustments are shown in Figure 9.

**FIGURE 10:**
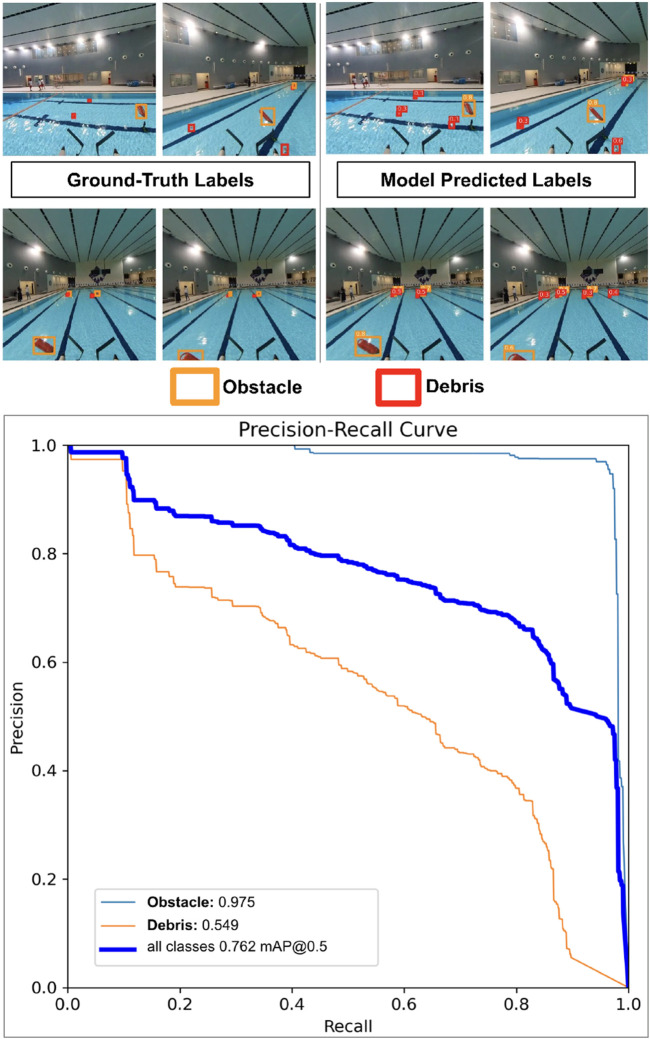
Model validation and training results.

The data were trained on YOLOv11 using the ADAM optimizer with an initial learning rate of 0.001, utilizing a cosine decay learning rate scheduler with three warmup epochs. A batch size of 16 was used, and the dataset contained 4,000 random images split into a 70–20–10 distribution for training, validation, and testing, respectively. Validation results are shown in Figure 10 along with the training results. The model achieves an mAP50 of 0.76 and an mAP95 of 0.365, with the precision-recall curves shown in Figure 10 (bottom). Overall, in sea states of 3 and above with poor lighting conditions, YOLO efficiently identified buoys (98% accuracy) but had several false positives recognized as cans (74% accuracy).

**FIGURE 11:**
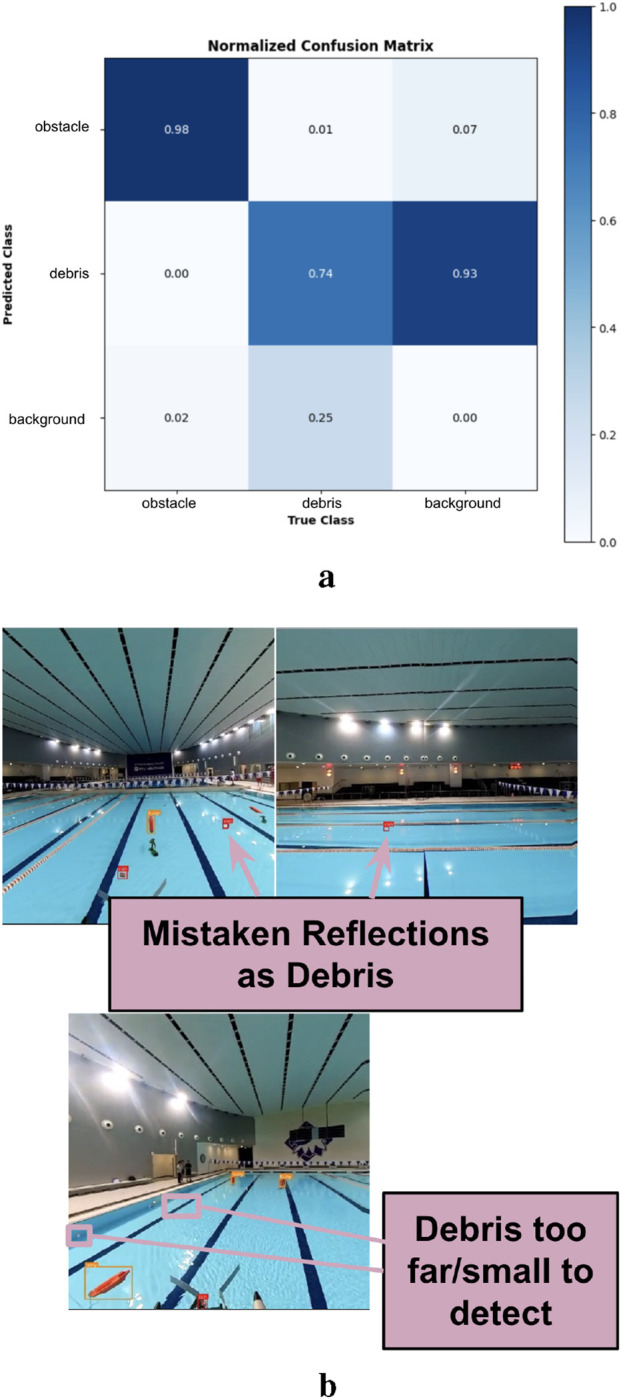
Confusion matrix and object detection failure cases. **(a)** Normalized Confusion Matrix, **(b)** Detection Failure Cases.

This model was further investigated using the confusion matrix shown in Figure 11a. Several false positives/negatives occurred in detecting debris (due to their small size) from the background (poor lighting conditions); several samples are shown in Figure 11b. The false positives were primarily due to the sharp reflections in the water surface (environmental conditions), while the false negatives occurred when the debris appeared small in the images. Notably, a) the superclass debris had several classes under it; these classes included cans, plastics, and others, and b) the summation of column-elements in the normalized confusion matrix should be one. The confusion matrix implies that the buoys were classified and identified correctly, the debris was recognized with a 74% accuracy, and the small cases of background were misclassified as debris (93% of instances). This inference, when using a GPU RTX 4050 GPU, resulted in a 25-ms delay.

**FIGURE 12:**
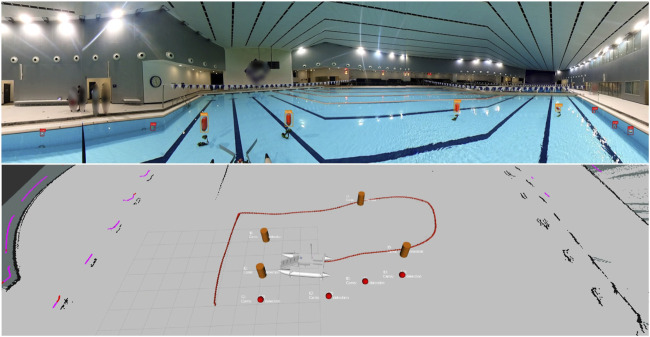
Stitched and annotated left, front, right, and back image views inferred with the YOLOv11 model (top) are used to locate debris and obstacles in the 2D map (bottom).

The model is inferred from the left, front, right, and back image views, as shown in Figure 12, where buoys are represented by orange cylinders and cans are represented by red markers (bottom). Furthermore, Figure 12 showcases a successful 2D-to-3D projection using the bounding box coordinates to estimate the position of various debris and obstacles.

### 4.3 USV kinodynamic measurements

The data sampling period was 20 ms, while $\omega^{\max}=0.3\;rad/s$ . These were measured from the onboard IMU’s gyroscope readings during rapid rotational maneuvers.

Since no direct velocity sensor was available, the maximum linear velocity was estimated by numerically integrating the acceleration data obtained from the IMU’s accelerometer. The USV started from a stationary position and accelerated until it reached terminal velocity, $v^{\max}=1.47m/s$ . The control signal on the resultant ATRON thrust and torque is nearly identical for the clockwise and counterclockwise rotation of propellers.

### 4.4 Odometry and sensor fusion

**FIGURE 13:**
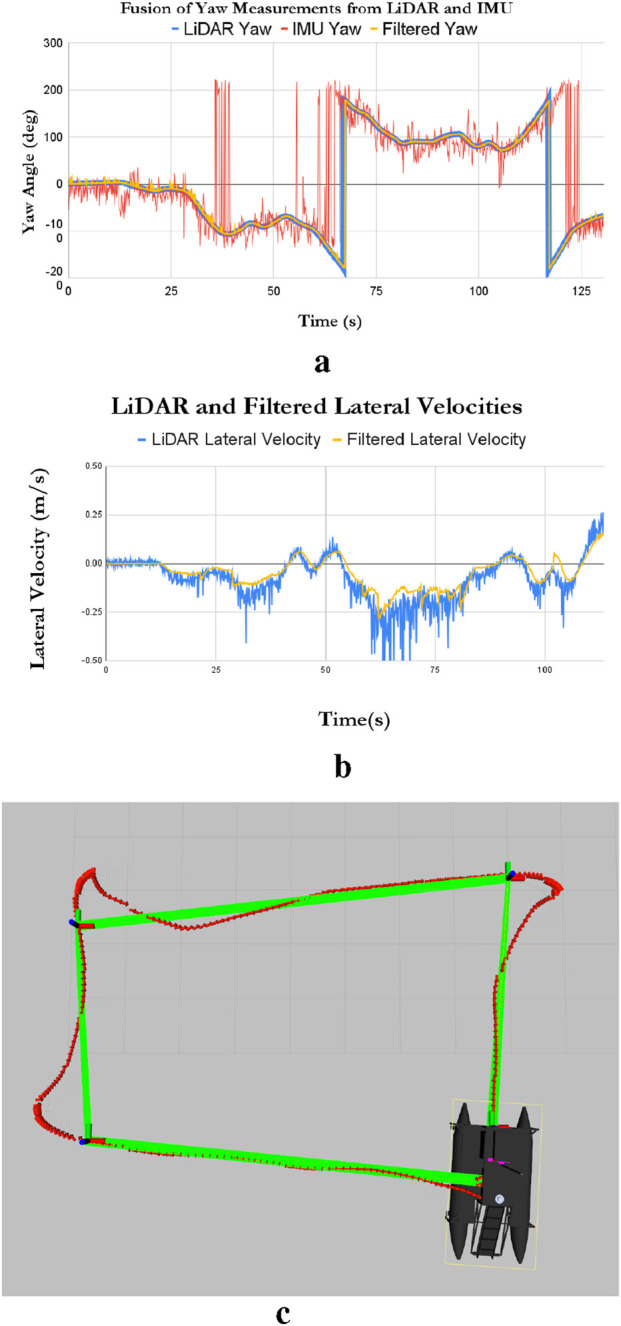
Experimental USV results: **(a)** LiDAR fusion with IMU generating yaw angles. **(b)** USV lateral velocities. **(c)** Experimental USV path following.

An EKF was used to fuse LiDAR data with IMU using the robot localization technique (Censi, 2008). Figure 13a shows the yaw angles obtained from LiDAR odometry, from the IMU, and from the fused data. The EKF fuses the smooth LiDAR odometry (blue) with the noisy IMU orientation (red) to produce a robust and accurate fused orientation (yellow). Notably, inclusion of the IMU is necessary since the USV can have small pitch and roll angles because of the sea state (waves and currents).

The USV’s lateral velocity is shown in Figure 13b, reflecting tilting of the USV.

### 4.5 Path following results

To test the path-following capabilities of the USV, a path consisting of four corners was to be followed. The USV’s actual experimental response is shown in Figure 13c, where the use of the PID-based control significantly improves the robot’s path-following capabilities by reducing its overshoot.

Automatic tuning (Åström et al., 1993) was used for obtaining the PID parameters. The ultimate gain $K_{u}$ and ultimate period $T_{u}$ were different for the linear and angular (torsional) components of the USV-controller: for the linear controller, $K_{u}=5$ and $T_{u}=2$ s, while for the angular controller, $K_{u}=3$ and $T_{u}=2$ s. The PID parameters used were

$$K_{p}=\frac{K_{u}}{3},\;K_{i}=\frac{2K_{u}}{3T_{u}},\;K_{d}=\frac{K_{u}T_{u}}{9}.$$

### 4.6 Path tracking for various sea states

**FIGURE 14:**
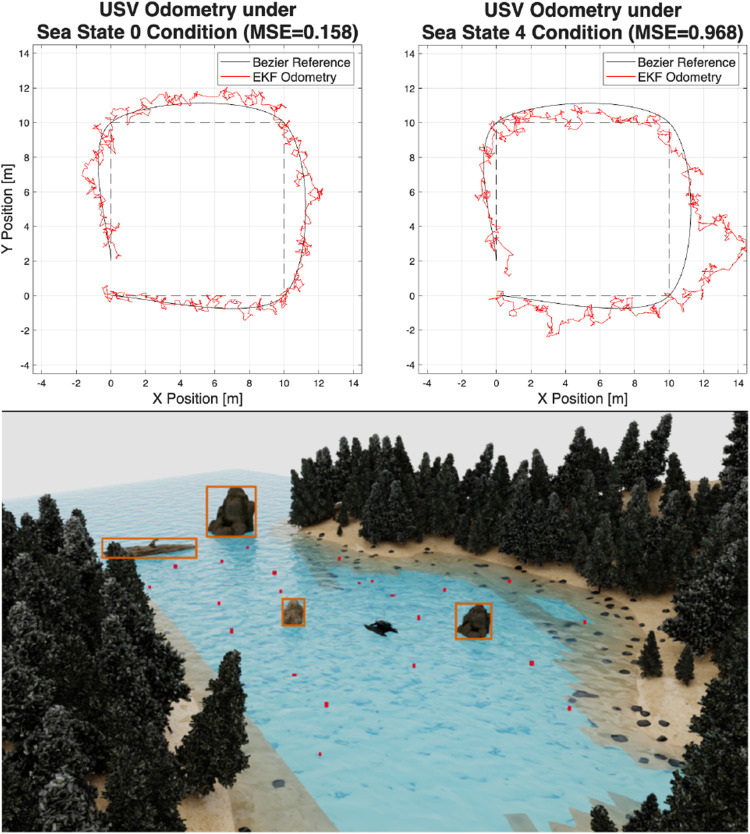
USV odometry tracking performance for different sea states (top) within the improved simulated environment (bottom).

The remote environment used in the simulation studies is shown at the bottom of Figure 14. This environment was used in NIVIDA’s Isaac simulator (Collins et al., 2021); this simulates the USV’s buoyancy, along with all sensors and transducers on top of the USV under ROS. Different sea state levels from 0 to 4 on the Beaufort wind scale are emulated (Meaden et al., 2007), which involves waves up to 4 m. Furthermore, the induced wind and the roll and pitch angles are nonzero and affect the effectiveness of the 2D LiDAR, the odometry system, and the ability to recognize the debris. The varying angles create erroneous behavior in the MRPP algorithm (Macenski et al., 2023), resulting in rapid turns, as shown in the upper half of Figure 14, while tracking a square 10×10 m. In both cases, Bezier smoothing (Arvanitakis and Tzes, 2012) (passing through the vertices of this square) was utilized to improve the tracking performance. The maximum attainable velocities were enforced during the simulation while different sea states were applied (Meaden et al., 2007).

## 5 Discussion

### 5.1 Summary of the results

This article presented ATRON, a large-scale autonomous USV designed for the removal of floating debris. With its 2.7×1.5 m footprint and 1 ${\text{m}}^{3}$ collection capacity, ATRON represents a significant advancement over existing small-scale prototypes, demonstrating the feasibility of practical autonomous marine cleanup systems.

The system’s key innovations include the novel use of a 360° camera with projection techniques for debris localization, avoiding the limitations of depth cameras in aquatic environments. The dual fisheye to equirectangular projection relies on placing the camera at an *a priori* known height. YOLOv11L successfully estimated debris positions, while the mapping capabilities were obtained with a 2D LiDAR. The integration of OMPL with the orienteering problem enabled optimal path planning, with the clustered variant effectively reducing computational complexity while maintaining collection efficiency.

Experimental validation in both an indoor pool environment and a simulated environment confirmed the system’s capabilities. The sensor fusion approach combining LiDAR odometry with IMU data through an extended Kalman filter provided robust localization despite the pitch and roll variations common in aquatic environments. The MRPP controller with PID control delivered precise path following, thus validating the overall system architecture.

### 5.2 Limitations and concluding remarks

Despite the demonstrated effectiveness of ATRON in controlled conditions, several limitations must be acknowledged. The USV has thus far only been evaluated in an indoor pool environment where hydrodynamic perturbations are negligible. While the catamaran’s low center of mass provides inherent passive stability, the absence of trials in open-water conditions with currents, waves, and wind precludes the validation of performance under realistic operating scenarios due to the necessary regulatory constraints and permit requirements.

A further limitation concerns the visual localization pipeline. The 2D-to-3D projection requires approximately 0.13 s per frame for calibration, which, at an angular velocity of 0.3 rad/s and an object distance of 10 m, introduces a positional differential of approximately 0.45 m. In addition, the absence of damping on the camera mount transmits vibrations from the thrusters and conveyor mechanism to the sensor, introducing noise into the projection parameters. This combination of calibration latency and vibration-induced disturbance degrades the accuracy of debris localization.

Future work will focus on addressing these limitations. The image-processing pipeline is being optimized through reduced resolution and refined projection algorithms to decrease calibration latency. Image stabilization is under development using both software- and hardware-based approaches, including the utilization of the integrated gyroscope in the 360° camera and the addition of physical damping elements. Furthermore, open-water trials are planned once regulatory requirements are satisfied, enabling the assessment of ATRON’s stability and performance in dynamic marine environments and advancing the system toward operational deployment.

## Data Availability

Publicly available datasets were analyzed in this study. These data can be found at https://github.com/RISC-NYUAD/ATRON.
